# Development and Validation of a Risk Score for Predicting Invasive Candidiasis in Intensive Care Unit Patients by Incorporating Clinical Risk Factors and Lymphocyte Subtyping

**DOI:** 10.3389/fcimb.2022.829066

**Published:** 2022-04-27

**Authors:** Jiahui Zhang, Wei Cheng, Dongkai Li, Jianwei Chen, Guoyu Zhao, Hao Wang, Na Cui

**Affiliations:** ^1^ Department of Critical Care Medicine, State Key Laboratory of Complex Severe and Rare Diseases, Peking Union Medical College Hospital, Chinese Academy of Medical Science and Peking Union Medical College, Beijing, China; ^2^ Department of Critical Care Medicine, Beijing Jishuitan Hospital, Beijing, China

**Keywords:** invasive candidiasis, risk prediction, critical care, lymphocyte subtyping, CD8+ T cell

## Abstract

**Objective:**

To develop and validate a rapid invasive candidiasis (IC)-predictive risk score in intensive care unit (ICU) patients by incorporating clinical risk factors and parameters of lymphocyte subtyping.

**Methods:**

A prospective cohort study of 1054 consecutive patients admitted to ICU was performed. We assessed the clinical characteristics and parameters of lymphocyte subtyping at the onset of clinical signs of infection and their potential influence on IC diagnosis. A risk score for early diagnosis of IC was developed and validated based on a logistic regression model.

**Results:**

Sixty-nine patients (6.5%) had IC. Patients in the cohort (N=1054) were randomly divided into a development (n=703) or validation (n=351) cohorts. Multivariate logistic regression identified that CD8+ T-cell count ≤143 cells/mm^3^, receipt of high-dose corticosteroids (dose ≥50 mg prednisolone equivalent), receipt of carbapenem/tigecycline, APACHE II score≥15, (1,3)-β-D-glucan (BDG) positivity and emergency gastrointestinal/hepatobiliary (GIT/HPB) surgery were significantly related with IC. IC risk score was calculated using the following formula: CD8+ T-cell count ≤143 cells/mm^3^ + receipt of high-dose corticosteroids + receipt of carbapenem/tigecycline + APACHE II score ≥15 + BDG positivity + emergency GIT/HPB surgery ×2. The risk scoring system had good discrimination and calibration with area under the receiver operating characteristic (AUROC) curve of 0.820 and 0.807, and a non-significant Hosmer-Lemeshow test P=0.356 and P=0.531 in the development and validation cohorts, respectively. We categorized patients into three groups according to risk score: low risk (0-2 points), moderate risk (3-4 points) and high risk (5-7 points). IC risk was highly and positively associated with risk score (Pearson contingency coefficient=0.852, P for trend=0.007). Candida score had a moderate predicting efficacy for early IC diagnosis. The AUROC of the risk score was significantly larger than that of Candida score (0.820 versus 0.711, Z=2.013, P=0.044).

**Conclusions:**

The predictive scoring system, which used both clinical factors and CD8+ T cell count, served as a clinically useful predictive model for rapid IC diagnosis in this cohort of ICU patients.

**Clinical Trial Registration:**

chictr.org.cn, identifier ChiCTR-ROC-17010750.

## Introduction

Critically ill patients are particularly vulnerable to invasive candidiasis (IC), and approximately one-third of all candidemia occurs in intensive care unit (ICU) (Delaloye and Calandra, 2014; Poissy et al., 2020). More importantly, IC-related outcomes remain poor, particularly if the initiation of antifungal therapy is delayed >12-24 hours after the first ultimately positive culture is drawn (Playford et al., 2016). However, initiation of culture-directed therapy within this window is difficult in practice, as culture systems generally do not signal positive for yeast until 48-72 hours of incubation. Timely antifungal intervention strategies, including prophylactic, preemptive, or empiric approaches, have therefore been increasingly advocated.

Given the low incidence of IC and the need to ensure appropriate and timely antifungal therapy, some form of risk stratification for IC becomes imperative to optimally separate patients at high risk of IC from those at low risk. This is especially important for those critically ill patients with IC at the onset of infection, because clinicians rarely consider an IC diagnosis in the ICU setting. Although several clinical factors and biomarkers such as (1,3)-β-D-glucan (BDG) are being investigated, they are not likely to prove useful in early diagnosis of IC at the onset of infection in ICU patients. T cells are part of the adaptive immune system and are responsible for the generation of immunological memory against invading pathogens. Our previous research demonstrated that massive loss of CD8+ T cells occurs at the onset of IC, and is closely associated with diagnosis in ICU patients, suggesting the utility of lymphocyte subtyping as a rapid testing tool with high value in early diagnosis of IC (Zhang et al., 2019).

Although several risk-predictive models based on clinical factors and/or Candida colonization parameters have been derived (Shorr et al., 2009; Playford et al., 2016), they have not all been well validated in patient cohorts outside their derivation population. This limits their generalizability. Furthermore, they have not been successful in quickly dichotomizing patients into high- and low-risk groups. Therefore, there is an unmet need for the early recognition of critically ill patients who may be at high risk of IC. In this study, we hypothesized that risk prediction scoring for the early diagnosis of IC at the onset of infection might be improved by incorporating potential risk factors and lymphocyte subtyping. This prospective study in ICU patients was conducted to develop and validate a scoring system for the early prediction of IC.

## Materials and Methods

### Study Design and Patients

The study was approved by the human research ethics committee of Peking Union Medical College Hospital (PUMCH, approval number: JS-1170) and adopted a prospective cohort study design. Consecutive patients admitted to PUMCH between March 2017 and June 2021 were selected according to the following inclusion criteria: (1) aged ≥18 years; (2) requirement for intensive care; (3) expected duration of stay in ICU >48 hours; and (4) clinical signs of infection as diagnosed by the attending physician meeting at least two of the following clinical criteria: (i) body temperature≥38°C or <36°C; (ii) respiratory rate≥30 breaths/min; (iii) pulse rate ≥120 beats/min; or (iv) abnormal total peripheral white blood cell count ≥10,000/mm^3^ or <4,000/mm^3^, or immature neutrophils>15% (Zhang et al., 2019). The diagnosis of IC should be made by experienced clinicians, according to the clinical signs of infection and at least one of the following criteria: (i) histopathological, cytopathological or direct microscopic confirmation of yeast cells in a specimen obtained by needle aspiration or biopsy from a normally sterile site (other than mucous membranes); (ii) at least one peripheral blood culture positive for Candida; (iii) positive Candida culture from a sample obtained by sterile technique from a normally sterile site (e.g. cerebrospinal, pleural, peritoneal or peritoneal abscess fluid) (De Pauw et al., 2008; Guo et al., 2013). We excluded (i) patients who were pregnant or lactating, (ii) patients ﻿with neutropenia (absolute neutrophil count <500/mm^3^) at baseline, (iii) patients with fungal infections other than those caused by Candida species, or (iv) patients who died within 48 hours.

### Data Collection

Clinical data included baseline demographics, comorbidities and the presence of recognized risk factors for IC. ﻿The risk factors for IC are exposure to broad-spectrum antibiotics, receipt of high-dose corticosteroids, acute physiology and chronic health evaluation (APACHE) II score≥15, BDG positivity, emergency gastrointestinal/hepatobiliary (GIT/HPB) surgery, dialysis, parenteral nutrition, and the use of CVCs (Pappas et al., 2016). The duration of corticosteroids was at least 3 weeks (De Pauw et al., 2008). Peripheral blood samples were collected at onset of infection for lymphocyte subtyping and related parameters. Lymphocyte subtyping and immunological parameters were determined from measurements of peripheral blood samples immediately by PUMCH laboratories. Peripheral blood mononuclear cells were separated, stained with different fluorescent monoclonal antibodies, then analyzed using flow cytometry (3-color EPICSXL flow cytometer; Beckman Coulter, Brea, California) to detect T cells (CD3+), CD4+ T cells (CD4+CD3+ and CD28+CD4+), CD8+ T cells (CD8+CD3+, CD28+CD8+), B cells (CD19+), and natural killer cells (CD3-CD16+CD56+). Monitoring was conducted by an independent clinical research organization to ensure implementation of good clinical practice in compliance with Chinese government regulations.

### Statistical Analysis

Descriptive statistics are reported as mean and standard deviation or median and interquartile range (IQR) for quantitative measures and counts and percentages for qualitative variables. Significance testing of comparisons was performed using parametric or nonparametric methods as appropriate. Baseline risk factors were calculated from measurements taking at the onset of infection. The dataset of 1054 patients were randomly divided into development (n=703) and validation (n=351) cohorts at a 2:1 ratio. The development cohort was used to create a risk score system, which was validated in the validation cohort. Logistic regression was used to identify potential characteristics associated with IC. Covariates of the final model were assigned weighted points calculated using a linear transformation of the corresponding ﻿β coefficient (divided by the smallest ﻿β coefficient and rounded to the nearest integer). A risk score was calculated for individual patients in the cohort. Patients were categorized as low, moderate, and high risk for IC based on tertiles of the risk score. The model discrimination was determined by the area under the receiver operating characteristic (AUROC) curve. Calibration of the model was determined by the Hosmer-Lemeshow goodness-of-fit test, with a non-significant value indicating good calibration. The final model was validated in the validation cohort. All the analyses were performed using ﻿SPSS version 22.0 (SPSS, Chicago, IL, USA) and R 3.4.4 (R Foundation for Statistical Computing, Vienna, Austria). A two-sided P-value <0.05 was considered statistically significant.

## Results

### Characteristics of the Development and Validation Cohorts

**Figure 1:**
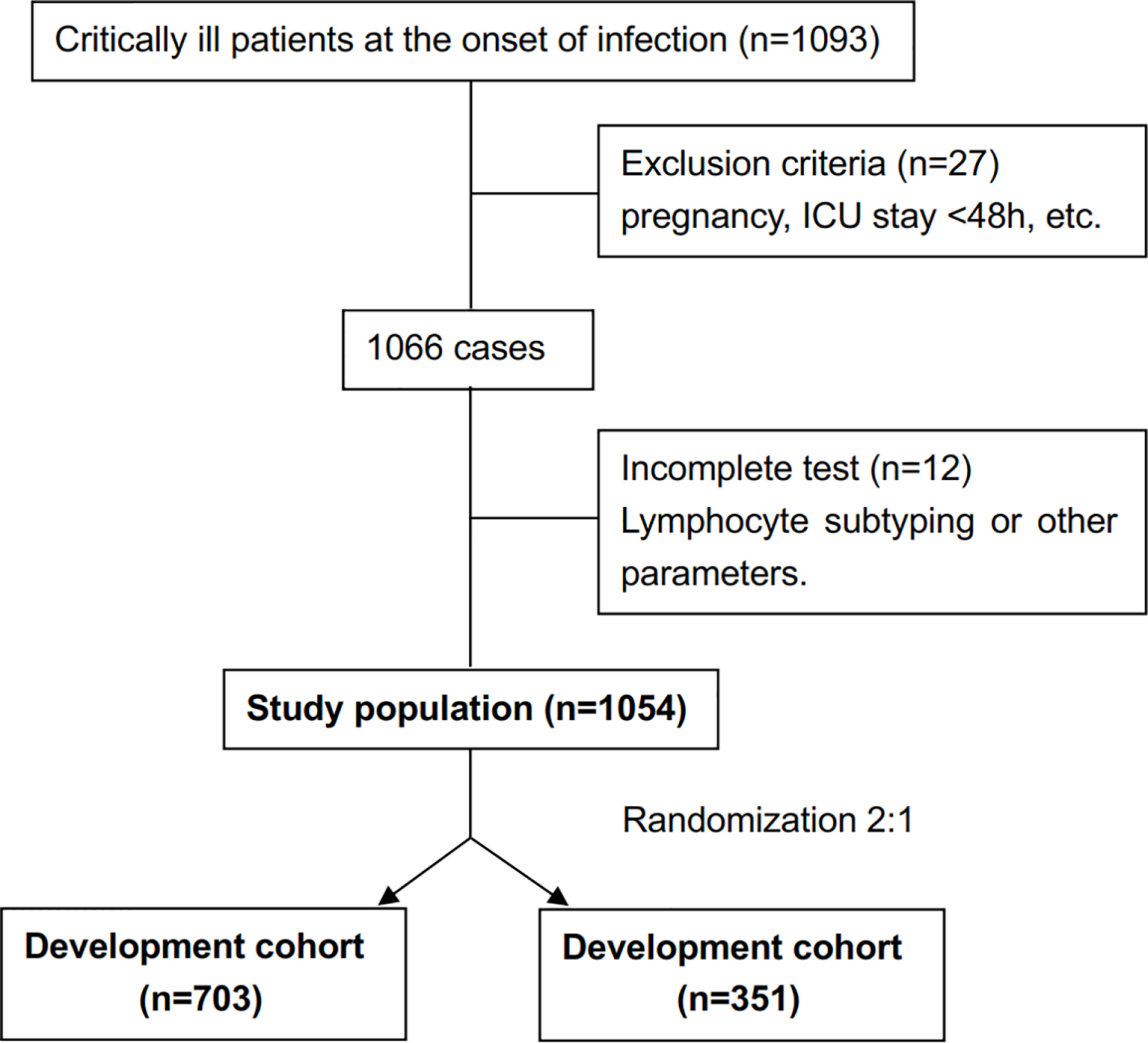
Flow diagram detailing the selection of patients included in the cohort.

The study cohort enrolled 1054 patients (**Figure 1**). The development cohort included 703 patients and the validation cohort included 351 patients. **Supplemental Table 1** shows the demographic and clinical characteristics of the two cohorts. There was no significant difference between the cohorts in terms of demographics, clinical characteristics, immune parameters and rates of IC. The incidence of IC was 6.5% (69/1054 patients).

### Risk Score Development

In the development cohort, potential risk factors associated with IC were identified by univariable logistic regression (**Table 1**). A previous study by our research group demonstrated that the cutoff of CD8+ T lymphocyte counts for predicting IC was 143 cells/mm^3^ (Zhang et al., 2019). We then added the variable of CD8+ T-cell counts ≤143 cells/mm^3^ to the multivariate logistic regression analysis. Variables independently associated with an increased risk of IC by multivariate logistic analysis included CD8+ T-cell counts ≤143 cells/mm^3^, receipt of high-dose corticosteroids (dose≥50mg prednisolone equivalent), receipt of carbapenem/tigecycline, acute physiology and chronic health evaluation (APACHE) II score≥15, BDG positivity and emergency gastrointestinal/hepatobiliary (GIT/HPB) surgery. The risk model constructed by the six variables had good discriminative power with a c statistic of 0.825 (95% confidence interval [CI] 0.760-0.890, P<0.001). The weighted points were assigned to each factor of the final model proportionally to their regression coefficients (**Table 2**). The risk score for IC was calculated based on the following formula: CD8+ T-cell count ≤143 cells/mm^3^ + receipt of high-dose corticosteroids + receipt of carbapenem/tigecycline + APACHE II score≥15 + BDG positivity + emergency GIT/HPB surgery ×2. Almost 64.7% of patients had risk scores of 0-2 (**Table 3**). The median score for patients with and without IC was 4 (IQR 2) and 2 (IQR 2), respectively (P<0.001).

**Table 1:** Demographic and clinical characteristics of the development cohorts at onset of infection according to IC diagnosis (N=703).

Variables	No IC (n=657, 93.5%)	IC (n=46, 6.5%)	*P* value*
Mean age (y)	62 (49-70)	66 (57-69)	0.096
Gender, male	410 (62.4)	33 (71.7)	0.133
**Underlying disease, n (%)**			
COPD	42 (6.4)	1 (2.2)	0.209
Diabetic mellitus	169 (25.7)	12 (26.1)	0.538
Chronic renal failure	65 (9.9)	7 (15.2)	0.180
Hepatic failure	24 (3.7)	4 (8.7)	0.103
Solid tumor	120 (18.3)	12 (26.1)	0.133
Immune system disease	39 (5.9)	5 (10.9)	0.152
Hematological disease	66 (10.0)	5 (10.9)	0.505
APACHE II score≥15	396 (60.3)	39 (84.8)	0.001
SOFA score>8	397 (60.4)	29 (63.0)	0.426
**Infection marker at the onset of signs of infection**			
PCT level (ng/ml)	1.7 (0.5-6.6)	3.3 (0.6-10.4)	0.149
BDG positive	170 (25.9)	22 (47.8)	0.002
CRP level (mg/L)	95.2 (39.7-177.6)	124.6 (51.6-198.8)	0.332
**Life-sustaining treatments, n(%)**			
Need for mechanical ventilation	602 (91.6)	43 (93.5)	0.462
Need for vasopressor	577 (87.8)	42 (91.3)	0.640
Need for RRT	125 (19.0)	10 (21.7)	0.698
**Indwelling catheter, n(%)**			
Urinary catheter	647 (98.5)	46 (100.0)	0.399
CVC	605 (92.1)	45 (97.8)	0.244
**Drug therapy, n(%)**			
High-dose corticosteroids receipt	128 (19.5)	21 (45.7)	<0.001
IVIG	33 (5.0)	2 (4.3)	0.839
Carbapenem/Tigecycline	315 (47.9)	33 (71.7)	0.002
Beta-lactam/beta-lactamase inhibitor combination	355 (54.0)	28 (60.9)	0.444
3rd/4th generation cephalosporin	131 (19.9)	13 (28.3)	0.187
**Other risk factors**			
Total parenteral nutrition, n(%)	88 (13.4)	17 (37.0)	<0.001
Emergency GIT/HPB Surgery, n(%)	55 (8.4)	15 (32.6)	<0.001
**Immune parameters**			
WBC (cells/mm3)	11710 (7400-16720)	11855 (7960-16410)	0.887
LY (cells/mm3)	875 (523-1326)	780 (522-930)	0.078
NK (cells/mm3)	66 (30-117)	61 (27-101)	0.530
LB (cells/mm3)	123 (57-207)	82 (43-178)	0.099
CD3+T (cells/mm3)	621 (347-978)	556 (331-615)	0.101
CD4+T (cells/mm3)	368 (213-571)	274 (147-404)	0.081
CD8+T (cells/mm3)	193 (98-336)	112 (84-175)	<0.001

APACHE II, Acute Physiology and Chronic Health Evaluation II; BDG, (1,3)-β-D-glucan; COPD, chronic obstructive pulmonary disease; CRP, C-reactive protein; CVC, central venous catheter; GIT/HPB, gastrointestinal/hepatobiliary; IVIG, intravenous immunoglobulin; PCT, procalcitonin; RRT, renal replacement therapy; SOFA, Sequential Organ Failure Assessment.

*P value for the comparison between no IC and IC groups.

**Table 2:** Multiple logistic regression model and weighted point assignment.

Variables	β	*P* value	Exp (β)	Exp (β)(95%CI)	Weighted points
CD8^+^ T-cell counts ≤143 cells/mm^3^	1.059	0.003	2.883	1.434-5.797	1
High-dose corticosteroids receipt	0.999	0.005	2.715	1.362-5.414	1
Carbapenem/Tigecycline	0.740	0.044	2.096	1.022-4.300	1
APACHE II score≥15	1.091	0.014	2.978	1.260-7.037	1
BDG positive	0.863	0.012	2.370	1.207-4.654	1
Emergency GIT/HPB Surgery	1.701	0.005	5.482	1.667-18.026	2
Total parenteral nutrition	0.345	0.542	1.412	0.466-4.282	—

**Table 3:** Distribution of the risk scores stratified by IC diagnosis in the development cohort.

Predictive score value	No IC (n=657, 93.5%)	IC (n=46, 6.5%)	Total (N=703)
0	59 (9.0)	1 (2.2)	60 (8.5)
1	161 (24.5)	1 (2.2)	162 (23.0)
2	227 (34.6)	6 (13.0)	233 (33.1)
3	127 (19.3)	10 (21.7)	137 (19.5)
4	57 (8.7)	15 (32.6)	72 (10.2)
5	25 (3.8)	5 (10.9)	30 (4.3)
6	1 (0.2)	7 (15.2)	8 (1.1)
7	0 (0.0)	1 (2.2)	1 (0.1)

Data are presented as No. (%).

### Validation of the Risk Score

**Figure 2:**
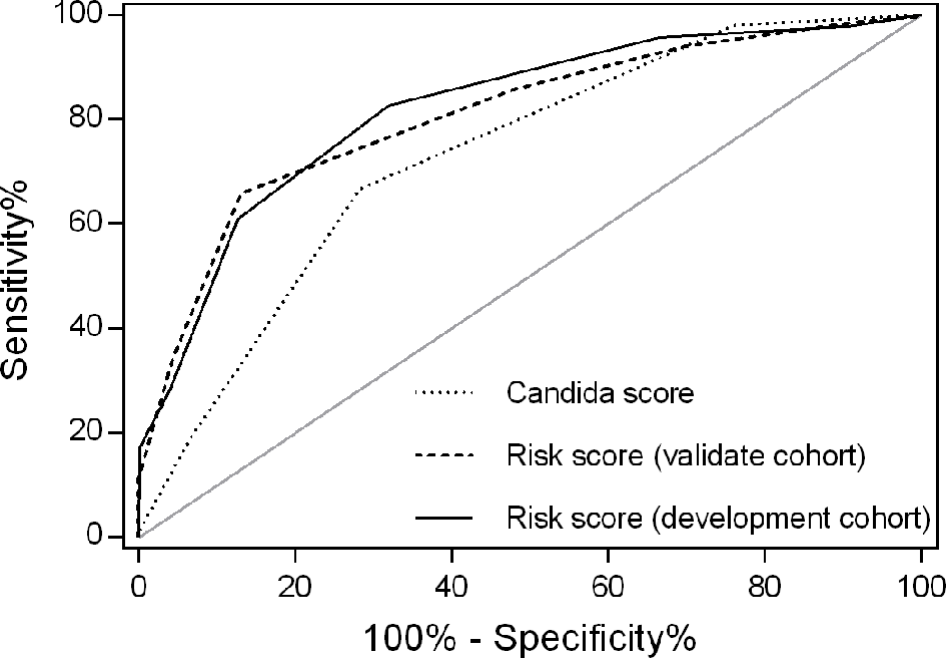
Receiver operating characteristics for IC risk score by cohort. The area under the receiver operating characteristic (AUROC) curve was 0.820 for the development cohort and 0.807 for the validation cohort. AUROCs were similar in both the development and validation cohorts (Z=0.236, P=0.813). However, the AUROC for the risk score was larger than that of *Candida* score (0.820 versus 0.711, P=0.044) in the development cohort.

**Figure 3:**
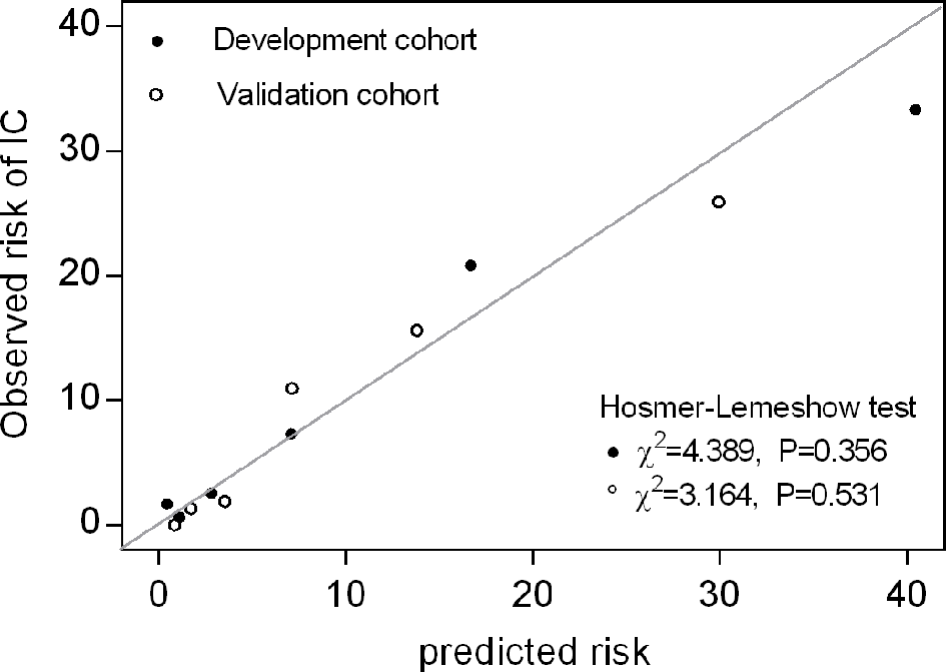
Calibration plot of observed versus predicted risk for IC at the onset of infection. Hosmer-Lemeshow chi-squared statistic is shown for the risk score in both the development and validation cohorts. The points and circles indicate the observed frequencies by sextile of predicted probability.

**Figure 4:**
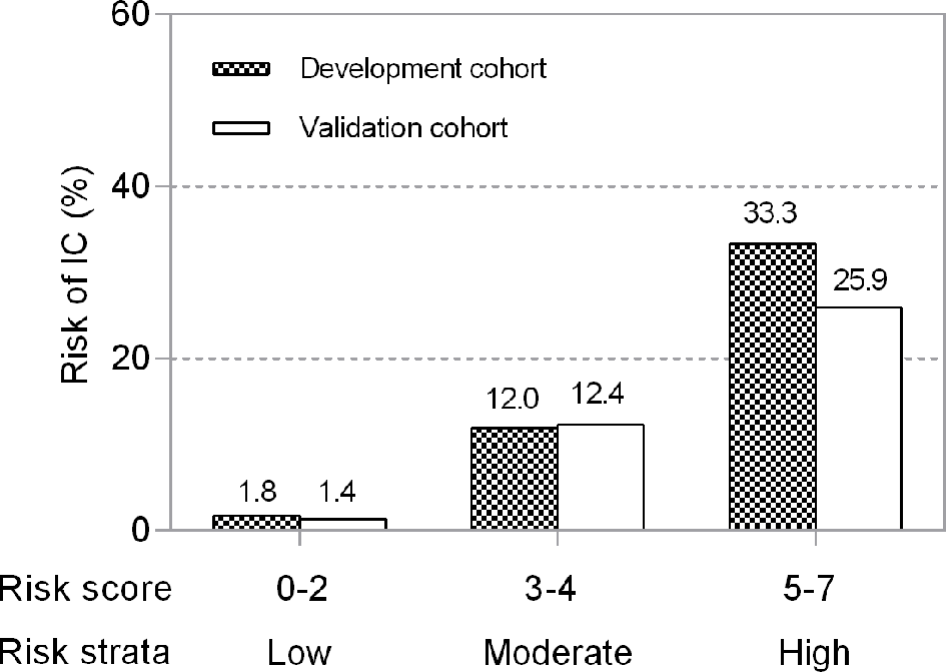
Risk levels according to the risk score in development and validation cohorts. Risks were categorized into low risk (0–2 points), moderate risk (3–4 points) and high risk (5–7 points). Higher points denote a higher risk of developing IC in ICU patients (P for trend=0.007).

In an independent validation cohort, the c statistic of the IC risk score was 0.807 (95% CI 0.726-0.889, P<0.001). The AUROCs were similar in both the development and validation cohorts (Z=0.236, P=0.813). The ROCs of the risk score are shown in **Figure 2**. The score had good calibration in both the development and validation cohorts with non-significant Hosmer-Lemeshow Chi-squares of 9.607 (P=0.294) and 5.111 (P=0.746), respectively (**Figure 3**). The rates of IC from both the development and validation cohorts were also concordant (**Figure 4**).

### Clinical Implications of the Risk Score

We divided the risk scores into three groups, i.e., low risk (0-2 points), moderate risk (3-4 points) and high risk (5-7 points), according to the tertiles of estimated risk, to enhance the clinical utility of the risk score. As shown in **Figure 3**, the risk of IC was highly and positively associated with the risk scores (Pearson contingency coefficient=0.852, P for trend=0.007). Patients in the low-risk group (0-2 points) comprised 64.7% of the entire cohort and 17.4% of IC cases; the incidence of IC in the group was 1.8%. Patients in the moderate-risk group (3-4 points) comprised 29.7% of the entire cohort and 54.3% of IC cases; the incidence of IC in the group was 12.0%. Patients in the high-risk group (5-7 points) comprised 5.5% of the entire cohort and 28.3% of IC cases; the incidence of IC in the group was 33.3%. The threshold of ≥5 resulted in a positive predictive value for IC of 33.3% (**Table 4**). In our study population, the sensitivity and specificity of BDG positivity for diagnosing IC were 47.8% and 74.1%, respectively. The best cut-off of the risk score was 3, with the sensitivity of 82.6% and specificity of 68% (**Table 4**). The sensitivity and specificity of the risk score were better than those of BDG positivity.

**Table 4:** Performance characteristics of risk-predictive model at different predictive score thresholds.

Characteristic	Predictive score threshold						
	≥1	≥2	≥3	≥4	≥5	≥6	≥7
Percentage of cohort ≥ threshold	91.5	68.4	35.3	15.8	5.5	1.3	0.1
Sensitivity, %	97.8	95.7	82.6	60.9	28.3	17.4	2.2
Specificity, %	9.0	33.5	68.0	87.4	96.0	99.9	100.0
Positive predictive value, %	7.0	9.1	15.3	25.2	33.3	88.9	100.0
Negative predictive value, %	98.3	99.1	98.2	97.0	95.0	94.5	93.6
Likelihood ratio for positive test	1.1	1.4	2.6	4.8	7.1	114.3	—
Likelihood ratio for negative test	0.24	0.13	0.26	0.45	0.75	0.83	0.98

### Performance Characteristics of Candida Score in the Study Cohort

A high Candida score was related with increased risk for IC (León et al., 2009), although Candida score had a moderate predicting efficacy for early diagnosis of IC (AUROC 0.711, P<0.001; **Figure 2**). The AUROC of the risk score was significantly larger than that of Candida score (0.820 versus 0.711, Z=2.013, P=0.044).

## Discussion

IC is an important complication of critical illness and is associated with considerable morbidity, mortality, and higher healthcare costs (Eggimann et al., 2003). Furthermore, delays in initiation of antifungal therapy are associated with increased mortality (Kollef et al., 2012). Conversely, over-prescription of antifungal therapy may be detrimental, exposing patients to drug toxicities and driving the emergence of resistance (Azoulay et al., 2012). Early diagnosis of IC is important but hampered by nonspecific clinical features, relatively slow turnaround times for culture results, and practical difficulties in sampling possible foci of infection in deep tissue. New diagnostic biomarkers such as BDG are not yet universally available, and have been reported to yield false-positive results (Usami et al., 2002; Marty et al., 2006). Considering these factors, strategies for timely antifungal therapy targeted to ICU patients at high risk for IC have been advocated (Pappas et al., 2009).

Several risk models to predict IC among ICU patients have been reported. Most research is based on retrospective analyses, and some models are based on risk factors at more than 24 hours or even several days after ICU admission (Shorr et al., 2009; Harrison et al., 2013; Playford et al., 2016). Furthermore, many studies focus only on candidemia, whereas deep-seated candidiasis also accounts for a large proportion of IC. We have performed this prospective cohort study of ICU patients to develop and validate a risk scoring system to identify patients at high risk of IC. As described, a wide range of clinical risk factors and parameters of lymphocyte subtyping were collected at the onset of infection to derive a model to predict IC. By multivariate logistic regression, six independent determinants of IC were identified. They comprised CD8+ T-cell count, use of high-dose corticosteroids, use of carbapenem/tigecycline, APACHE II score, BDG positivity and emergency GIT/HPB surgery. All six risk factors have been proved in previous reports. Broad-spectrum antibiotic use disrupts the skin and gut microbiome, increasing Candida colonization and the risk of IC (Eggimann and Pittet, 2014). In published meta-analyses of BDG studies, the pooled sensitivity and specificity for diagnosing IC were 75%-80% and 80%, respectively (Karageorgopoulos et al., 2011; Lu et al., 2011; Onishi et al., 2012). However, a number of issues complicate the interpretation of these data, including uncertainties about the best cutoff value for a positive result, number of positive tests required to establish a diagnosis, and optimal timing and frequency of testing among at-risk patients. In our study population, the sensitivity and specificity of the risk score for diagnosing IC were better than those of BDG positivity. We are aware that the sensitivity and specificity of the risk score were slightly lower and believe that future large-scale prospective multi-center study is needed to confirm our results. Intra-abdominal candidiasis accounts for most deep-seated IC, with about 30% of cases occurring in the ICU. Perforation, anastomotic leaks, repeat laparotomies and necrotizing pancreatitis increase risk. As a result, accumulating evidence shows that emergency gastrointestinal or hepatobiliary surgical procedures are closely related with a higher risk of IC (Bassetti et al., 2015; Playford et al., 2016). Recent *in vitro* and *in vivo* studies in humans and animals have pointed to the development of CD8+ T-cell exhaustion in IC (Spec et al., 2016; Guinault et al., 2021). We have previously demonstrated that CD8+ T immunity is impaired in response to IC through the mammalian target of rapamycin signaling pathway, and that the decreased CD8+ T-cell-count could predict IC (Zhang et al., 2021). The reports provide evidence that CD8+ T cell-count, which is quickly available through lymphocyte subtyping, is important for the early diagnosis of IC in ICU patients in clinical practice. Although there have been reports demonstrating that all six risk factors are significantly associated with diagnosis of IC, current evidence shows that each factor alone is not enough to identify patients at high risk of IC and to trigger the use of antifungal therapy (Logan et al., 2020). Because all six factors can be obtained quickly and easily, a predictive model that combines them, including clinical risk factors and lymphocyte subtyping, could be a promising tool for early IC diagnosis. The regression model was translated into a risk-predictive model by weighting each variable by its associate coefficient. The model performed reasonably well, with a Harrel C score of 0.820. Candida score is commonly used for IC diagnosis in ICU populations (León et al., 2009). However, it requires the results of fungal cultures. Results of these cultures can take at least two days to obtain, which does not assist in timely diagnosis. In the present study, the risk score had a larger AUROC for IC diagnosis than that of Candida score. This suggests that the risk score had a better diagnostic performance for early recognition of IC.

The present study identified three groups according to estimated risk. First, the low-risk group (risk score 0-2 points) comprised 64.7% of the cohort and had a very low incidence of IC (1.8%). In clinical practice this group would not be initiated on antifungal therapy. Second, the high-risk group (risk score 5-7 points) accounted for 5.5% of the cohort and had a very high incidence of IC (33.3%). This group might benefit from initiation of antifungal therapy. The remaining group with moderate risk (risk score 3-4 points), accounted for 29.7% of the entire cohort and had a 12% incidence of IC. Patients in this group might require additional assessment, and the need for antifungal therapy is not clear-cut. Additional testing, for example, the use of next-generation sequencing, might be of benefit in this group. Future studies are needed to confirm the role of this risk score in initiating or discontinuing antifungal therapy.

There are several limitations to this study. First, our risk-score model contains more variables than others, potentially posing practical difficulties for translation to the bedside. However, all variables in the model are readily available and could contribute to early assessment of the risk of IC. Second, the study cohort comprised a relatively heterogeneous group of patients, including both medical and surgical patients. However, this reflects the real-life scenario in many ICU settings. Finally, this is a single-center study, and a large-scale prospective multi-center study is needed to confirm our results.

## Conclusions

The predictive scoring system, which used both clinical factors and CD8+ T cell-counts, may serve as a clinically useful predictive model for the rapid diagnosis of IC in ICU patients. This rapid risk-score model that stratifies those at risk for IC, may help clinicians to quickly rule out IC or to identify patients at higher risk for this disease at the onset of infection.

## Data Availability Statement

The original contributions presented in the study are included in the article/**Supplementary Material**. Further inquiries can be directed to the corresponding authors.

## Ethics Statement

The studies involving human participants were reviewed and approved by the human research ethics committee of Peking Union Medical College Hospital. The patients/participants provided their written informed consent to participate in this study.

## Author Contributions

NC and HW came up with the idea and polished the article. JZ performed the data analysis and the writing of the article. WC and DL contributed to the data analysis. JC and GZ contributed to the data collection. All authors contributed to the article and approved the submitted version.

## Funding

The work was supported by National Natural Science Foundation of China [No. 82072226], Beijing Municipal Science and Technology Commission [No. Z201100005520049], Tibet Natural Science Foundation [No. XZ2019ZR-ZY12(Z)], and Excellence Program of Key Clinical Specialty of Beijing in 2020 [No. ZK128001].

## Conflict of Interest

The authors declare that the research was conducted in the absence of any commercial or financial relationships that could be construed as a potential conflict of interest.

## Publisher’s Note

All claims expressed in this article are solely those of the authors and do not necessarily represent those of their affiliated organizations, or those of the publisher, the editors and the reviewers. Any product that may be evaluated in this article, or claim that may be made by its manufacturer, is not guaranteed or endorsed by the publisher.
