# Supplementary material for: Development and Validation of a Risk Score for Predicting Invasive Candidiasis in Intensive Care Unit Patients by Incorporating Clinical Risk Factors and Lymphocyte Subtyping
Source: Front Cell Infect Microbiol. 2022 Apr 27;12:829066. doi: 10.3389/fcimb.2022.829066 (PMC9091371; doi:10.3389/fcimb.2022.829066)
Supplement: Supplementary file 1 [file Table_1.docx]

Supplemental Table 1 Demographic and clinical characteristics of the development and validation cohorts

| **Variables** | **Development cohort**  **(N=703)** | **Validation cohort**  **(N=351)** | ***P* value*** |
| --- | --- | --- | --- |
| Mean age (y) | 63 (52-71) | 66 (49-73) | 0.127 |
| Gender, male | 443 (63.0) | 227 (64.7) | 0.635 |
| **Underlying disease, n (%)** |  |  |  |
| COPD | 43 (6.1) | 15 (4.3) | 0.253 |
| Diabetic mellitus | 181 (25.7) | 84 (23.9) | 0.547 |
| Chronic renal failure | 72 (10.2) | 46 (13.1) | 0.132 |
| Hepatic failure | 28 (4.0) | 7 (2.0) | 0.102 |
| Solid tumor | 132 (18.8) | 78 (22.2) | 0.118 |
| Immune system disease | 44 (6.3) | 32 (9.1) | 0.100 |
| Hematological disease | 71 (10.1) | 32 (9.1) | 0.613 |
| APACHE Ⅱ score≥15 | 435 (61.9) | 209 (59.5) | 0.503 |
| SOFA score>8 | 426 (60.6) | 206 (58.7) | 0.100 |
| **Infection marker at the onset of signs of infection** | | | |
| PCT level (ng/ml) | 1.73 (0.5-5.8) | 1.40 (0.3-5.9) | 0.124 |
| BDG positive | 192 (27.3) | 77 (21.9) | 0.061 |
| CRP level (mg/L) | 96.8 (40.5-180.1) | 104.2 (47.7-195.8) | 0.543 |
| **Life-sustaining treatments, n(%)** | | | |
| Need for mechanical ventilation | 645 (91.7) | 304 (86.6) | 0.109 |
| Need for vasopressor | 619 (88.1) | 303 (86.3) | 0.102 |
| Need for RRT | 135 (19.2) | 71 (20.2) | 0.693 |
| **Indwelling catheter, n(%)** |  |  |  |
| Urinary catheter | 693 (98.6) | 350 (99.7) | 0.087 |
| CVC | 650 (92.5) | 321 (91.5) | 0.335 |
| **Drug therapy, n(%)** |  |  |  |
| High-dose corticosteroids receipt | 149 (21.2) | 75 (21.4) | 0.949 |
| IVIG | 35 (5.0) | 18 (5.1) | 0.917 |
| Carbapenem/Tigecycline | 348 (49.5) | 192 (54.7) | 0.117 |
| Beta-lactam/beta-lactamase inhibitor combination | 383 (54.5) | 185 (52.7) | 0.102 |
| 3rd/4th generation cephalosporin | 144 (20.5) | 86 (24.5) | 0.154 |
| **Other risk factors** |  |  |  |
| Total parenteral nutrition, n(%) | 105 (14.9) | 67 (19.1) | 0.086 |
| Emergency GIT/HPB Surgery, n(%) | 70 (10.0) | 49 (14.0) | 0.063 |
| **Immune parameters** |  |  |  |
| WBC (cells/mm3) | 11940 (7510-16925) | 10180 (6690-13620) | 0.103 |
| LY (cells/mm3) | 870 (501-1283) | 813 (451-1131) | 0.112 |
| NK (cells/mm3) | 65 (29-116) | 53 (27-96) | 0.068 |
| LB (cells/mm3) | 125 (55-205) | 112 (45-173) | 0.097 |
| CD3+T (cells/mm3) | 612 (336-967) | 542 (312-878) | 0.144 |
| CD4+T (cells/mm3) | 367 (204-564) | 331 (164-511) | 0.060 |
| CD8+T(cells/mm3) | 185 (93-334) | 182 (101-298) | 0.689 |
| **Rates of IC** | 46 (6.5) | 23 (6.6) | 0.995 |

APACHE Ⅱ, Acute Physiology and Chronic Health Evaluation Ⅱ; BDG, (1,3)-β-D-glucan; COPD, chronic obstructive pulmonary disease; CRP, C-reactive protein; CVC, central venous catheter; GIT/HPB, gastrointestinal/hepatobiliary; IVIG, intravenous immunoglobulin; PCT, procalcitonin; RRT, renal replacement therapy; SOFA, Sequential Organ Failure Assessment.

* *P* value for the comparison between development and validation cohorts.
